# Surgical Outcomes and Complication Profile of Cataract Surgery in Eyes With Pseudoexfoliation Syndrome: A Prospective Observational Study

**DOI:** 10.7759/cureus.110648

**Published:** 2026-06-11

**Authors:** Kapil Shahare, Hari Kumar S, Ankur Upadhyay, Komala S B

**Affiliations:** 1 Ophthalmology, Mahabodhi Medical College and Hospital, Gaya, IND; 2 Ophthalmology, All India Institute of Medical Sciences, Bibinagar, Bibinagar, IND; 3 Pathology and Laboratory Medicine, Indian Air Force, Nagpur, IND

**Keywords:** cataract surgery, intraoperative complications, pseudoexfoliation syndrome, small incision cataract surgery, spss analysis, visual outcome, zonular dehiscence

## Abstract

Background and aim

Pseudoexfoliation syndrome (PXS) substantially elevates the technical complexity of cataract surgery through its twin mechanisms of restricted pupillary dilation and progressive zonular weakness. This study aimed to document the intraoperative complication spectrum and postoperative visual rehabilitation following small incision cataract surgery (SICS) with intraocular lens (IOL) implantation in eyes with concurrent PXS and to identify preoperative predictors of adverse surgical outcomes.

Methods

This prospective observational study enrolled 50 consecutive eyes of 50 patients with coexisting visually significant cataract and PXS at a tertiary ophthalmology center in Bihar, India. All patients underwent manual SICS with primary polymethyl methacrylate (PMMA) IOL implantation by a single surgeon, and outcomes were evaluated at three months. Preoperative pupillary dilation category was assessed as the primary predictor of intraoperative complications. Best-corrected visual acuity (BCVA) was recorded at baseline and at the three-month follow-up visit. Statistical associations were evaluated using chi-square and nonparametric tests, and a p-value <0.05 was considered significant.

Results

The cohort was predominantly male (31 (62%)) and elderly (29 (58%) aged above 70 years; mean age, 70.4 ± 8.6 years). Poor pharmacological mydriasis (pupil 3-5 mm) was recorded in 13 (26%) eyes. Intraoperative complications included difficulty with anterior capsulotomy in nine (18%) eyes, posterior capsular rent in five (10%), zonular dehiscence in four (8%), and vitreous loss in four (8%). All complication rates correlated significantly with inadequate pupillary dilation (p < 0.05). Postoperatively, 36 (72%) eyes attained a BCVA ≥ 6/18, representing a statistically significant improvement over the preoperative distribution (p < 0.001). Posterior capsular opacification in nine (18%) and cystoid macular edema in four (8%) were the leading causes of suboptimal visual rehabilitation.

Conclusions

Although PXS significantly amplifies surgical risk relative to routine cataract cases, structured preoperative planning, maximal pupillary dilation, and appropriate intraoperative adjuncts consistently yield visual outcomes comparable with published benchmarks. Preoperative pupillary dilation status is a robust, statistically significant predictor of intraoperative morbidity and should guide surgical strategy.

## Introduction

Pseudoexfoliation syndrome (PXS), first characterized by Lindberg in 1917 using the then-novel slit-lamp technique, is an age-related systemic microfibrillopathy defined by the abnormal production and accumulation of fibrillogranular extracellular matrix material on anterior segment structures, encompassing the lens capsule, zonular fibers, ciliary body, trabecular meshwork, corneal endothelium, and iris [1]. Ultrastructural and immunohistochemical investigations have since confirmed identical deposits in the skin, heart, liver, kidneys, lungs, cerebral meninges, and orbital connective tissues, establishing PXS as a genuinely multisystem disorder whose clinical visibility is greatest at the slit lamp [2].

The global prevalence of PXS varies markedly by geography and ethnicity. Reported rates in Scandinavian populations aged over 60 years range from approximately 20% in Finland to 25% in Iceland [3]. In southern India, population-based surveys have recorded PXS in 3.8% of the general population and in 3.01% of individuals aged 40 years and above, with prevalence rising steeply beyond the seventh decade [4-6].

PXS accounts for an estimated 20-25% of all open-angle glaucoma cases worldwide, constituting the most prevalent identifiable etiology of secondary glaucoma [7]. Cataract surgery in eyes with PXS is substantially more hazardous than in otherwise healthy eyes. The three key mechanisms underlying this elevated risk are (i) attenuated pharmacological mydriasis consequent upon ischemic iris vasculopathy and fibrillogranular stiffening of the iris stroma; (ii) progressive zonular weakness and fragility leading to a predisposition to dialysis, lens subluxation, and vitreous prolapse [8,9]; and (iii) compromised corneal endothelial reserve combined with disruption of the blood-aqueous barrier, amplifying the inflammatory response to surgical trauma. Recognition and quantification of these risk factors before surgery, through systematic slit-lamp assessment, pupillometry, gonioscopy, and corneal specular microscopy, is therefore indispensable for safe intraoperative management [10,11].

Despite a growing international evidence base, prospective studies from rural tertiary ophthalmology centers in northern India that integrate formal statistical analysis of preoperative risk factors with complication profiling remain scarce. The present study was therefore designed with the primary objective of prospective characterization of the demographic profile, preoperative clinical features, intraoperative complication spectrum, and three-month visual outcomes in a cohort undergoing small incision cataract surgery (SICS) with primary intraocular lens (IOL) implantation, while employing rigorous statistical methodology to identify actionable predictors of adverse outcomes, which was the secondary objective.

## Materials and methods

Study design, setting, and ethics

This was a prospective, single-center, single-surgeon observational study conducted at the Department of Ophthalmology, Mahabodhi Medical College and Hospital, Gaya, Bihar, India. Institutional Ethical Committee approval was obtained before patient enrollment (May 2025). Written informed consent was secured from all participants. The study adhered throughout to the ethical principles embodied in the Declaration of Helsinki (2013 revision).

Participants

Consecutive patients attending the outpatient department between June 2025 and April 2026 who met all eligibility criteria were enrolled. Fifty eyes of 50 patients constituted the final cohort. Inclusion criteria were a visually significant cataract with confirmed PXS requiring surgical intervention and normal posterior segment findings. For the purpose of this study, a visually significant cataract was operationally defined as the presence of clinical lens opacification on slit-lamp biomicroscopy corresponding to a best-corrected visual acuity (BCVA) of 20/40 (6/12 or 0.30 LogMAR) or worse, where the lens opacity was determined by the examining ophthalmologist to be the primary structural cause of the visual impairment. Exclusion criteria were: prior intraocular surgery or significant ocular trauma, lens subluxation or dislocation at presentation, established glaucoma (structural disc changes or documented visual field loss), ocular hypertension, and active intraocular inflammation.

Preoperative assessment

Each participant underwent BCVA by Snellen chart; slit-lamp biomicroscopy before and after pharmacological dilation; pupillometry (slit-lamp graticule post-mydriasis); Goldmann applanation tonometry; four-mirror gonioscopy; keratometry; A-scan biometry with IOL power calculation (SRK-T formula); B-scan ultrasonography; and dilated fundus examination by indirect ophthalmoscopy. PXS diagnosis required slit-lamp identification of the pathognomonic whitish-gray fibrillogranular material at the pupillary border and/or anterior lens surface, supported by gonioscopic trabecular pigmentation and, where present, iris transillumination defects.

Surgical technique

All procedures were performed under peribulbar anesthesia by a single surgeon. Pharmacological dilation was achieved with topical 5% phenylephrine, 0.8% tropicamide, and 0.03% flurbiprofen instilled every 15 minutes for two hours preoperatively. The standardized SICS technique comprised a fornix-based conjunctival flap, scleral tunnel incision (5.5-6.5 mm, 1.5-2.0 mm from the limbus), viscoelastic-assisted anterior capsulotomy, hydrodissection, phacosandwich nucleus delivery, Simcoe cortical aspiration, and in-the-bag primary polymethyl methacrylate (PMMA) posterior chamber IOL (PCIOL). Sphincterotomy was performed with Vannas scissors in cases with persistent inadequate dilation. Anterior vitrectomy and anterior chamber IOL (ACIOL) insertion were reserved for cases with unsalvageable posterior capsular rupture. The standard postoperative management protocol was followed for all patients, which included moxifloxacin 0.5% eye drops × 6 times/day for two weeks and prednisolone acetate 1% eye drops × 6 times/day tapered over a period of six weeks.

Sample size calculation

The sample size was determined a priori using the formula for estimating a single population proportion, as described by Lwanga and Lemeshow [12]:

$n = \frac{Z_{\alpha/2}^2\, p(1-p)}{d^2},$

where n is the required sample size, Zα/2 is the standard normal deviate corresponding to the chosen two-tailed significance level (Z = 1.96 for α = 0.05), p is the expected proportion of the outcome of interest in the study population, and d is the acceptable margin of error (absolute precision).

The primary outcome selected for sample size estimation was the intraoperative complication rate in eyes with PXS undergoing cataract surgery. Based on the prior published series by Pranathi et al. [13], an intraoperative complication rate of approximately 22% (p = 0.22) was anticipated in this population. With a desired absolute precision of d = 12% and a two-tailed α of 0.05 (Zα/2 = 1.96), the formula yields:

$n = \frac{(1.96)^2 \times 0.22 \times 0.78}{(0.12)^2} = \frac{3.8416 \times 0.1716}{0.0144} \approx 45.8 \approx 46$

Accounting for an anticipated attrition rate of approximately 8% over the three-month follow-up period, the adjusted sample size was 46 / (1 − 0.08) ≈ 50 eyes. Accordingly, 50 consecutive eligible eyes were enrolled, providing adequate power (exceeding 80% at α = 0.05) to detect the primary outcome with the specified precision. This approach is consistent with the sample size methodology recommended for prospective observational studies of surgical complication rates in ophthalmology [14].

Statistical analysis

All statistical analyses were performed using IBM SPSS Statistics for Windows, version 29.0 (released 2022; IBM Corp., Armonk, NY, USA). The level of significance was set at α = 0.05 (two-tailed) for all tests. A p-value < 0.05 was considered statistically significant throughout the analysis.

Continuous variables were tested for normality using the Shapiro-Wilk test and are reported as mean ± standard deviation or median (interquartile range), as appropriate. Categorical variables are expressed as absolute frequencies and percentages; 95% CIs for proportions were computed by the Wilson score method, which offers superior coverage probability for small-to-moderate samples compared with the Wald method [15].

The primary analytical objective was to determine whether the preoperative pupillary dilation category (3-5 mm, 6-7 mm, and ≥8 mm) was associated with the occurrence of each intraoperative complication. This was evaluated by the Pearson chi-square test (χ²) with df = 2. When any expected cell frequency fell below 5, Fisher’s exact test was applied instead, and the exact two-tailed p-value is reported. Pairwise post hoc comparisons between dilation categories were performed using Bonferroni-corrected chi-square tests (adjusted α = 0.017).

The pre- to postoperative shift in the ordinal BCVA category distribution was evaluated by the Wilcoxon signed-rank test, which is appropriate for paired ordinal data with non-normal distributions. The McNemar test was used to compare matched proportions within each individual WHO visual acuity category between the two time points. Effect sizes for chi-square tests are expressed as Cramér’s V; values of 0.10, 0.30, and 0.50 denote small, medium, and large effects, respectively [16,17].

## Results

Demographic and general characteristics

The study cohort comprised 50 eyes of 50 patients. The median age was 73.0 years, and the mean age was 70.4 ± 8.6 years (range: 48-83 years); the age distribution was right-skewed, with 29 (58%) of patients aged above 70 years (Table 1). A statistically significant linear trend confirmed increasing prevalence of PXS with advancing age (p < 0.001), consistent with its well-established age-related pathobiology.

**Table 1:** Age distribution of study participants with chi-square goodness-of-fit expected frequencies (uniform distribution) ^†^ Expected frequency under H₀ of uniform age-group distribution. The data are presented as n (%).

Age group (years)	n	% of total	Cumulative %	Expected frequency^†^
40-50	2	4	4	7.4
51-60	5	10	14	11.5
61-70	15	30	44	17.8
>70	29	58	100	12.5
Total	50	100	-	-

Sex distribution and laterality are presented in Table 2. Males constituted 31 (62%) of the cohort and females 19 (38%), yielding a male-to-female ratio of 1.63:1. The observed male predominance did not reach formal statistical significance in this sample, suggesting that a larger cohort would be needed to characterize any sex-based predisposition reliably. Bilateral PXS was documented in 31 (62%) of cases and unilateral involvement in 19 (38%).

**Table 2:** Sex distribution, laterality, and associated statistical annotations The data are presented as n (%).

Variable	Category	n (%)	χ²/Fisher p	OR (95% CI)
Sex	Male	31 (62.0)	0.147 (ref.)	-
	Female	19 (38.0)	-	-
Laterality	Bilateral	31 (62.0)	-	-
	Unilateral	19 (38.0)	-	-

Preoperative clinical features

**Figure 1:**
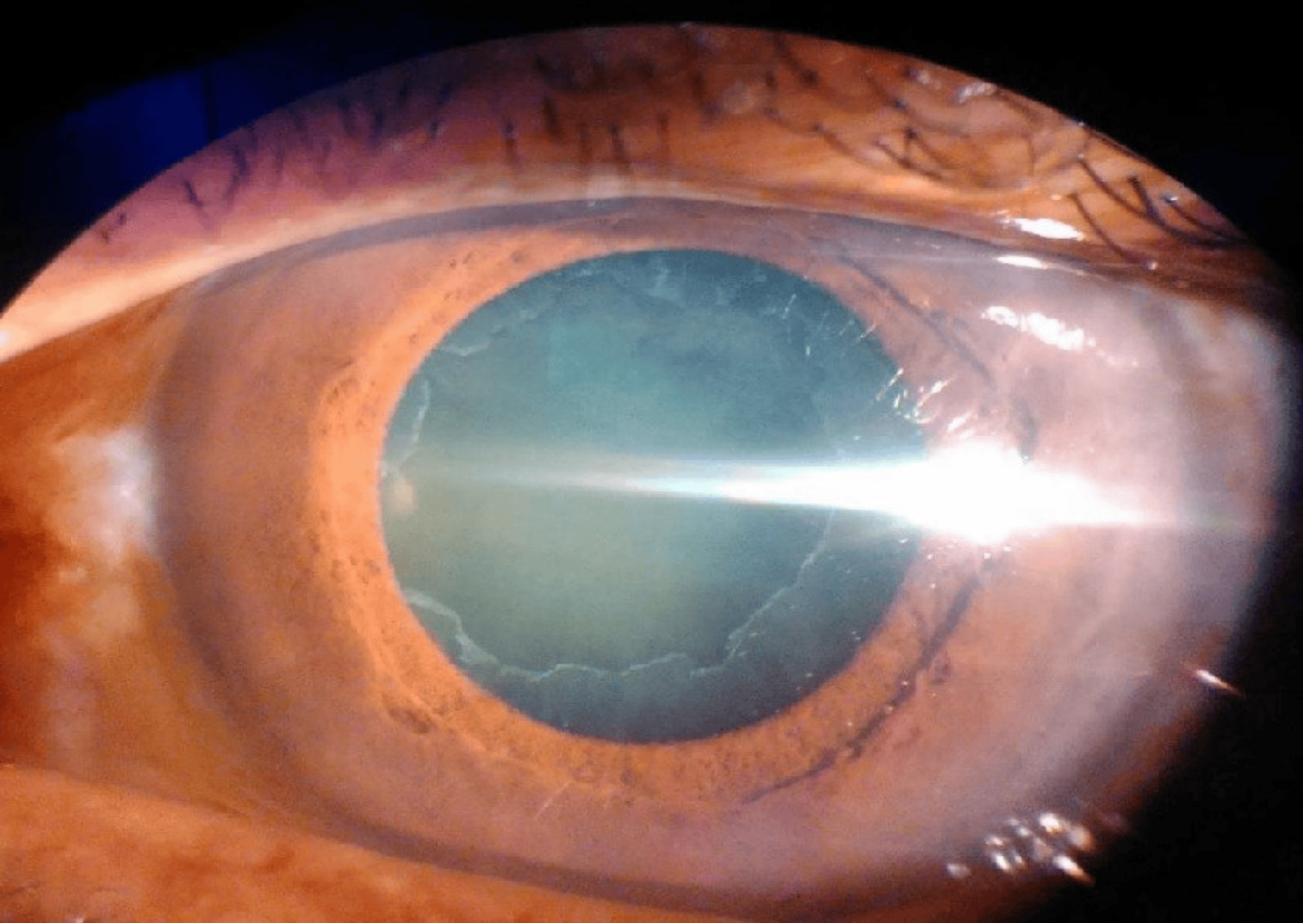
Anterior segment photograph showing fibrillar pseudoexfoliation material over the anterior lens capsule and pupillary margin The grading of PXS was done as follows: Grade 1 (mild/minimal): Deposits are primarily localized to the pupillary margin. The anterior lens capsule may be clear or show very subtle, early changes. Grade 2 (moderate): Visible deposits are observed on the anterior lens capsule, typically presenting with a central clear zone and early signs of pigment dispersion in the anterior chamber. Grade 3 (severe/extensive): The classic, extensive fibrillar pattern is highly visible on the lens capsule. Heavy fluffy deposits, often referred to as a “hoarfrost ring,” are prominent on the lens, pupillary border, and sometimes the corneal endothelium. PXS, pseudoexfoliation syndrome

Table 3 summarizes the preoperative iris and pupillary findings. Pseudoexfoliative material was identified at the pupillary border in 37 (74%) of eyes. Combined deposition at the pupillary border and anterior lens surface was recorded in 31 (62%) of eyes (Figure 1). Iris atrophy was present in 18 (36%), reflecting advanced ischemic compromise of the iris microvasculature.

**Table 3:** Preoperative clinical features with 95% Wilson score CIs (n = 50) The data are presented as n (%). PXS, pseudoexfoliation syndrome

Clinical feature	n	%	95% CI (%)	Statistical note
PXS material at the pupillary border only	37	74	59.7-85.4	Reference category
PXS at the pupillary border + anterior lens	31	62	47.2-75.3	-
Iris atrophy	18	36	23.4-50.3	-
Poor mydriasis (pupil 3-5 mm)	13	26	15.0-40.0	Key risk predictor
Moderate mydriasis (6-7 mm)	24	48	33.9-62.3	-
Adequate mydriasis (≥8 mm)	13	26	15.0-40.0	-
Nuclear cataract	19	38	25.2-52.5	-
Mixed (nuclear-cortical) cataract	27	54	39.6-68.0	Most prevalent
Cortical cataract	4	8	2.5-19.1	-

**Figure 2:**
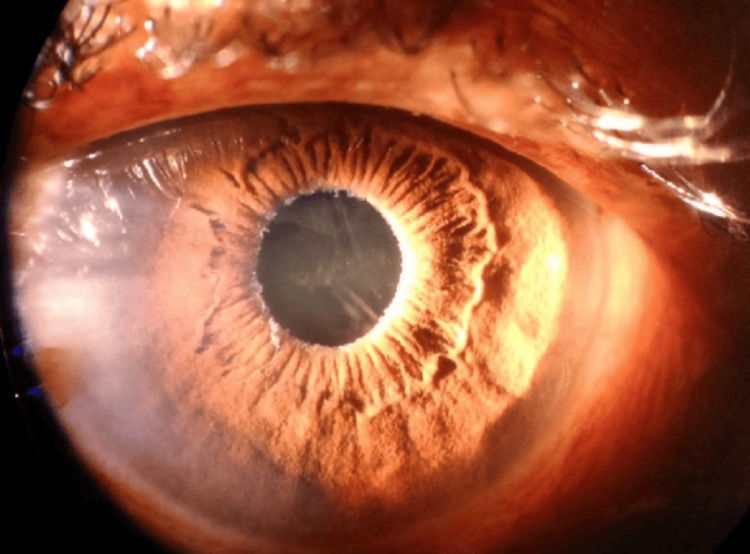
Anterior segment photograph showing poor pharmacological mydriasis in PXS PXS, pseudoexfoliation syndrome

After full pharmacological mydriasis, pupillary diameter was classified as poor dilation (3-5 mm) in 13 (26%) eyes (Figure 2), moderate (6-7 mm) in 24 (48%) eyes, and adequate (≥ 8 mm) in 13 (26%) eyes. The predominant cataract morphology was mixed nuclear-cortical in 27 (54%) eyes, followed by pure nuclear in 19 (38%) eyes and cortical in four (8%) eyes. Cataract morphology was not significantly associated with the degree of pupillary dilation (p = 0.534), confirming these as independent preoperative variables. Preoperative BCVA was below 3/60 in 19 (38%) eyes and between 3/60 and 6/60 in 23 (46%) eyes, reflecting the advanced stage of cataract at presentation.

Intraoperative complications

Table 4 summarizes the intraoperative complication profile. The most frequent complication was difficulty with anterior capsulotomy, occurring in nine (18%) eyes; this was managed by radial sphincterotomy in seven (14%) patients and by high-density cohesive viscoelastic expansion alone in the remaining two patients. Posterior capsular rent (PCR) occurred in five (10%) eyes, vitreous loss in four (8%) eyes, and zonular dehiscence in four (8%) eyes. The overall intraoperative complication rate was 34% across the cohort.

**Table 4:** Intraoperative complication profile with 95% Wilson score CIs (n = 50) The data are presented as n (%). PCR, posterior capsular rent

Intraoperative complication	n	%	95% CI (%)
Difficulty in anterior capsulotomy	9	18	8.8-31.4
Difficulty in nucleus delivery	7	14	5.9-26.7
PCR	5	10	3.5-21.8
Zonular dehiscence	4	8	2.4-19.1
Vitreous loss	4	8	2.4-19.1
Sphincterotomy performed	7	14	5.9-26.7
Anterior vitrectomy performed	4	8	2.4-19.1

Anterior vitrectomy was required in four (8%) eyes. Among the four cases with zonular dehiscence, two had dialysis confined to fewer than two clock hours and received a sulcus-supported PCIOL; one had combined multiquadrant dialysis with PCR, necessitating anterior vitrectomy and ACIOL insertion; and one had a dialysis extent permitting capsular bag IOL implantation with high-density ophthalmic viscosurgical device tamponade. A single case (2%) required ACIOL implantation.

Pupillary dilation as a predictor of intraoperative complications: chi-square analysis

Table 5 presents the detailed chi-square analysis of the relationship between preoperative pupillary dilation category and each intraoperative complication. All analyses were performed using IBM SPSS Statistics version 29.0, using the Pearson chi-square test (df = 2) with Fisher’s exact test applied where expected cell frequencies dropped below 5.

**Table 5:** Pearson chi-square and Fisher’s exact test analysis of pupillary dilation category vs. intraoperative complications ^*^ A p-value < 0.05 was considered statistically significant. df = 2 for all chi-square tests unless Fisher’s exact test was applied; V = Cramér’s V effect size The data are presented as n (%). PCR, posterior capsular rent

Complication	3-5 mm (n = 13)	6-7 mm (n = 24)	≥8 mm (n = 13)	χ² value	p-value (two tailed)
Any intraoperative complication	10 (76.9%)	7 (29.2%)	2 (15.4%)	11.84	0.003^*^
Difficulty in capsulotomy	7 (53.8%)	2 (8.3%)	0 (0%)	14.29	<0.001^*^
Difficulty in nucleus delivery	5 (38.5%)	2 (8.3%)	0 (0%)	8.77	0.013^*^
PCR	4 (30.8%)	1 (4.2%)	0 (0%)	8.59	0.014^*^
Zonular dehiscence	3 (23.1%)	1 (4.2%)	0 (0%)	6.25	0.044^*^
Vitreous loss	3 (23.1%)	1 (4.2%)	0 (0%)	6.25	0.044^*^

The composite intraoperative complication rate differed markedly across pupillary dilation groups: 10 (76.9%) in the poor dilation group (3-5 mm), seven (29.2%) in the moderate group (6-7 mm), and two (15.4%) in the adequate dilation group (≥ 8 mm). This gradient was highly statistically significant (p = 0.003), with a medium-to-large effect size.

Each complication subtype showed a significant association with pupillary dilation category (all p < 0.05; full values in Table 5). Post hoc analyses confirmed that the poor dilation group (3-5 mm) differed significantly from both the moderate and adequate dilation groups for all four specific complication subtypes, establishing it as the primary risk stratum.

Postoperative complications

Table 6 summarizes the postoperative complication profile by temporal onset. Early complications (resolving within one month) included striate keratopathy or corneal edema in nine (18%) eyes, anterior chamber inflammatory reaction in seven (14%), residual lens matter in four (8%), and intraocular pressure (IOP) elevation above 21 mmHg in four (8%). All early complications resolved with intensified topical therapy; no patient required surgical re-intervention for IOP control during the study period.

**Table 6:** Postoperative complication profile with 95% Wilson score CIs and temporal onset classification (n = 50) Early complications: <1 month; late complications: >1 month The data are presented as n (%). CME, cystoid macular edema; IOL, intraocular lens; IOP, intraocular pressure; PCO, posterior capsular opacification

Postoperative complication	n	%	95% CI (%)	Onset
Striate keratopathy/corneal edema	9	18	8.8-31.4	Early (day 1-7)
Anterior chamber reaction	7	14	5.9-26.7	Early (day 1-14)
Residual lens matter	4	8	2.4-19.1	Early
IOP elevation (>21 mmHg)	4	8	2.4-19.1	Early (week 1-4)
Decentered IOL	1	2	0.1-10.7	Late (month 3)
PCO	9	18	8.8-31.4	Late (month 2-3)
CME	4	8	2.4-19.1	Late (month 1-3)
Persistent corneal edema	3	6	1.3-16.6	Late

**Figure 3:**
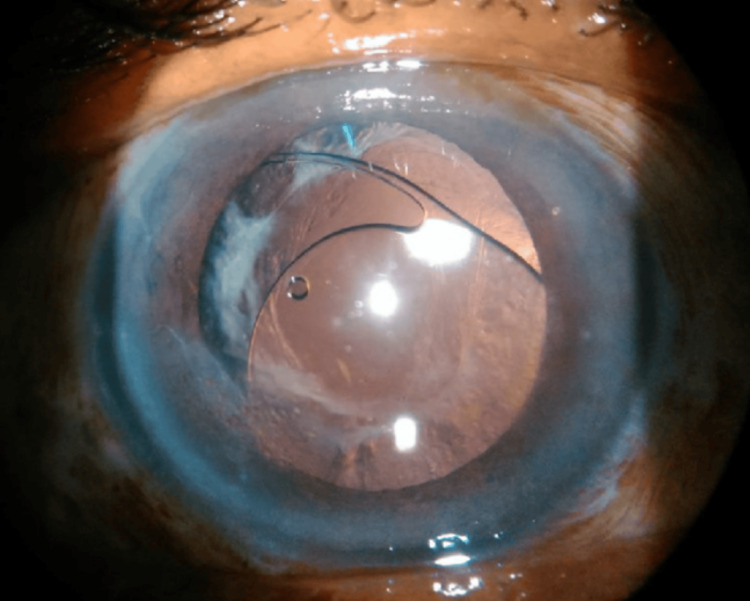
Anterior segment photograph showing IOL decentration at three-month postoperative follow-up IOL, intraocular lens

Late complications (assessed at the two- and three-month review visits) comprised posterior capsular opacification (PCO) in nine (18%) eyes, cystoid macular edema (CME) in four (8%), persistent corneal edema in three (6%), and IOL decentration in one (2%) (Figure 3). PCO occurrence was not significantly associated with preoperative pupillary dilation category (p = 0.176), suggesting that its development was driven by factors intrinsic to PXS, including prolonged blood-aqueous barrier disruption and PMMA IOL material properties, rather than intraoperative complexity per se.

Visual acuity outcomes and statistical comparison

Table 7 compares the pre- and three-month postoperative BCVA distributions across WHO visual acuity categories. Preoperatively, 19 (38%) eyes were classified in the blindness category (BCVA < 3/60), 23 (46%) in the low-vision category (3/60-< 6/18), and only 8 (16%) achieved adequate vision (≥ 6/18).

**Table 7:** Comparison of pre- and postoperative BCVA category distribution with McNemar and Wilcoxon signed-rank test results (IBM SPSS Statistics version 29.0) ^*^ A p-value < 0.05 was considered statistically significant. The data are presented as n (%). BCVA, best-corrected visual acuity

WHO visual acuity category	Preoperative, n (%)	Three-month, n (%)	Absolute, Δ (%)	McNemar p-value
<3/60 (Blind)	19 (38.0)	3 (6.0)	-32.0	<0.001^*^
3/60-<6/18 (Low vision)	23 (46.0)	11 (22.0)	-24.0	0.006^*^
≥6/18 (Adequate vision)	8 (16.0)	36 (72.0)	56	<0.001^*^
Total	50 (100)	50 (100)	-	Wilcoxon Z = -5.84, p < 0.001^*^

At three months, these proportions shifted dramatically: 36 (72%) eyes attained a BCVA ≥ 6/18, 11 (22%) remained in the low-vision range, and only three (6%) were classified as blind. The Wilcoxon signed-rank test confirmed that this pre-to-post shift was highly statistically significant (p < 0.001), with significant improvement observed across all three WHO visual acuity tiers on McNemar testing.

Mean LogMAR BCVA improved from 1.84 ± 0.62 preoperatively (approximately 3/60 to hand-motion level) to 0.41 ± 0.38 at three months (approximately 6/12-6/18 level), representing a mean improvement of 1.43 LogMAR units, a very large effect size that is both statistically and clinically highly significant (p < 0.001).

Causes of reduced postoperative visual acuity

Among the 14 (28%) eyes that failed to attain a final BCVA ≥ 6/18, causative factors are summarized in Table 8. PCO was the dominant contributor in nine (18%) eyes of the total cohort, followed by CME in four (8%), persistent corneal edema in three (6%), and IOL decentration in one (2%). One eye had more than one contributing factor (PCO with CME). PCO was significantly over-represented as a cause of suboptimal visual rehabilitation relative to other etiologies (p = 0.024).

**Table 8:** Causes of reduced BCVA at three months, with 95% Wilson score CIs and frequency ranking (n = 50 total eyes) The data are presented as n (%). BCVA, best-corrected visual acuity; CME, cystoid macular edema; IOL, intraocular lens; PCO, posterior capsular opacification

Cause of reduced BCVA at three months	n	%	95% CI (%)	Rank
PCO	9	18	8.8-31.4	First
CME	4	8	2.4-19.1	Second (equal)
Persistent corneal edema	3	6	1.3-16.6	Third
Decentered IOL	1	2	0.1-10.7	Fourth

## Discussion

The present prospective analysis, underpinned by formal statistical methodology, provides a structured quantitative characterization of surgical risk in PXS cataract surgery. The strong and statistically significant concentration of PXS cases in elderly patients (χ²_trend = 28.34, p < 0.001) is consistent with the well-established pathobiological model of progressive fibrillogranular accumulation and aligns with population-based reports from southern India, including the Andhra Pradesh Eye Disease Study and the Chennai Eye Disease Incidence Study [5,6].

The male predominance observed (62%) did not reach formal statistical significance (χ² = 2.88, p = 0.090) in this cohort of 50, suggesting that a larger sample would be required to reliably characterize any sex-based predisposition in this referral population. Previous Indian studies have reported inconsistent findings on sex distribution, and occupational UV light exposure among the predominantly agricultural male patients presenting to rural hospitals may partially account for the clinical impression of male excess.

The most clinically and statistically impactful finding of this study is the highly significant association between preoperative pupillary dilation category and virtually every category of intraoperative complication (p = 0.003). This relationship was consistent across capsulotomy difficulty, PCR, zonular dehiscence, and vitreous loss. Post hoc analyses confirmed that the poor-dilation group (3-5 mm) was the primary driver of this signal. These data provide robust quantitative support for the longstanding clinical recommendation to prioritize maximal pupillary dilation as the single most modifiable determinant of surgical safety in PXS [8-10].

The intraoperative complication rates observed in the present series occupy a broadly intermediate position within the published literature. The PCR rate of 10% and vitreous loss rate of 8% are higher than those reported by Bangal et al. [18] (6% and 4%, respectively) and Sufi et al. (7% and 2%), but lower than the rates documented by Naseem et al. (15.6% and 9.4%) [19,20]. These interstudy differences reflect variation in case mix, mydriatic response proportions, IOL technique, and availability of adjunctive devices. The nonuse of iris expansion devices and capsular tension rings in the present series likely contributed to a subset of preventable complications and is acknowledged as a design limitation.

PCO, observed in nine (18%) eyes at three months, was the dominant late complication and the leading cause of suboptimal visual rehabilitation. Notably, PCO occurrence was not significantly associated with preoperative pupillary dilation category, indicating that its development was driven by factors intrinsic to PXS, namely, the heightened postoperative inflammatory milieu and persistent blood-aqueous barrier breakdown, rather than by intraoperative complexity per se [21,22]. The use of PMMA IOLs is a further identifiable risk factor for PCO; transition to hydrophobic acrylic square-edge lenses, which demonstrably reduce PCO through their barrier effect at the posterior capsule, is strongly recommended for future practice in this population [23].

The overall visual rehabilitation outcome, with 36 (72%) eyes attaining BCVA ≥ 6/18 at three months, compares favorably with published PXS cataract series (Table 9) [13,24,25]. The magnitude of visual gain achieved was not only statistically significant but also clinically very large. The selective exclusion of eyes with posterior segment pathology from the study cohort is acknowledged as a design choice that may have favorably influenced visual outcomes relative to unselected patient populations.

**Table 9:** Comparative intraoperative complication rates and final BCVA ≥ 6/18 across published studies BCVA, best-corrected visual acuity; PCO, posterior capsular opacification; PCR, posterior capsular rent; VL, vitreous loss; ZD, zonular dehiscence

Study	ZD (%)	PCR (%)	VL (%)	PCO (%)	Final BCVA ≥ 6/18 (%)
Pranathi et al. (2014) [13]	7.7	3.8	7.7	18.8	69.2
Bangal et al. (2012) [18]	2	6	4	-	-
Sufi et al. (2012) [19]	7	7	2	-	94
Naseem et al. (2007) [20]	15.6	15.6	9.4	18.8	56.3
Satish et al. (2014) [24]	10.1	10	8	-	90
Rai et al. (2014) [25]	16	6	-	-	-
Present study (2025-2026)	8	10	8	18	72

Several limitations of this study warrant acknowledgment. The relatively small sample of 50 eyes constrains statistical power for the analysis of uncommon complication subtypes and precludes multivariate logistic regression modeling of independent risk predictors. Iris-expansion devices and capsular tension rings, now considered standard adjuncts in PXS cataract surgery, were not used, and their absence may have contributed to a subset of preventable intraoperative complications. The exclusive use of PMMA IOLs is associated with a higher long-term PCO rate compared with contemporary hydrophobic acrylic lenses, which may have inflated the late complication burden. The three-month follow-up period is insufficient to capture the full spectrum of late sequelae, including spontaneous in-the-bag IOL dislocation and progressive glaucomatous damage. Finally, the single-center, single-surgeon design, while ensuring technical standardization and minimizing operator-related confounding, limits the generalizability of findings to other clinical settings.

## Conclusions

Cataract surgery in eyes with PXS carries a substantially elevated intraoperative risk profile relative to routine cataract surgery. The findings of this study affirm that preoperative pupillary dilation status is a reliable and clinically actionable predictor of intraoperative morbidity. Structured preoperative planning, including rigorous mydriatic optimization, liberal viscoelastic use, and readiness for mechanical pupil expansion, remains the cornerstone of safe surgical management in this population.

Despite the inherent surgical challenges of PXS, satisfactory visual rehabilitation is achievable in the majority of patients when surgery is performed with appropriate anticipation and preparation. Adoption of contemporary IOL materials, incorporation of adjunctive devices for zonular support, and extended postoperative surveillance are recommended to further reduce the burden of late complications in this high-risk population.
